# The common oncogenomic program of NOTCH1 and NOTCH3 signaling in T-cell acute lymphoblastic leukemia

**DOI:** 10.1371/journal.pone.0185762

**Published:** 2017-10-12

**Authors:** Sung Hee Choi, Eric Severson, Warren S. Pear, Xiaole S. Liu, Jon C. Aster, Stephen C. Blacklow

**Affiliations:** 1 Department of Biological Chemistry and Molecular Pharmacology, Harvard Medical School, Boston, MA, United States of America; 2 Department of Cancer Biology, Dana Farber Cancer Institute, Boston, MA, United States of America; 3 Department of Pathology, Brigham and Women’s Hospital and Harvard Medical School, Boston, MA, United States of America; 4 Departments of Biostatistics and Computational Biology, Dana Farber Cancer Institute, and Harvard School of Public Health, Boston, MA, United States of America; 5 Department of Pathology and Laboratory Medicine, Abramson Family Cancer Research Institute, University of Pennsylvania Perelman School of Medicine, Philadelphia, PA, United States of America; Maastricht University, NETHERLANDS

## Abstract

Notch is a major oncogenic driver in T cell acute lymphoblastic leukemia (T-ALL), in part because it binds to an enhancer that increases expression of *MYC*. Here, we exploit the capacity of activated *NOTCH1* and *NOTCH3* to induce T-ALL, despite substantial divergence in their intracellular regions, as a means to elucidate a broad, common Notch-dependent oncogenomic program through systematic comparison of the transcriptomes and Notch-bound genomic regulatory elements of NOTCH1- and NOTCH3-dependent T-ALL cells. ChIP-seq studies show a high concordance of functional NOTCH1 and NOTCH3 genomic binding sites that are enriched in binding motifs for RBPJ, the transcription factor that recruits activated Notch to DNA. The interchangeability of NOTCH1 and NOTCH3 was confirmed by rescue of NOTCH1-dependent T-ALL cells with activated NOTCH3 and *vice versa*. Despite remarkable overall similarity, there are nuanced differences in chromatin landscapes near critical common Notch target genes, most notably at a Notch-dependent enhancer that regulates *MYC*, which correlates with responsiveness to Notch pathway inhibitors. Overall, a common oncogenomic program driven by binding of either Notch is sufficient to maintain T-ALL cell growth, whereas cell-context specific differences appear to influence the response of T-ALL cells to Notch inhibition.

## Introduction

Notch receptors play critical roles in metazoan development and cellular homeostasis. In normal signaling, receptor activation relies on *trans* interactions between Notch receptors and DSL (Delta, Serrate, and Lag2) ligands. Ligand binding stimulates receptor proteolysis, liberating the intracellular portion of Notch (ICN) from the membrane. ICN translocates to the nucleus where it forms a complex with the DNA-binding factor RBPJ and a transcriptional co-activator of the Mastermind-like family (MAML), stimulating transcription of Notch target genes [1, 2].

In mammals, there are four different Notch receptors and five DSL ligands. Each receptor has a similar domain organization, with a series of N-terminal, ligand-binding EGF-like repeats, followed by a negative regulatory region (NRR), a transmembrane segment, and an intracellular effector region, which includes a (RAM) region, seven iterated ankyrin (ANK) repeats, a transactivation domain (TAD), and a PEST domain [3]. Multiple sequence alignment shows that Notch1 and Notch2 are most similar, with divergence increasing in Notch3 and greatest in Notch4. The most highly conserved region of the four mammalian Notch proteins is the ankyrin repeat region, where there is 66% identity between NOTCH1 and NOTCH3. The region C-terminal to the ankyrin repeats, however, is much more divergent, with the transactivation domain (TAD) containing only 21% sequence identity. Deletion of the region encoding the Notch1 TAD in mice results in a hypomorphic phenotype with perinatal lethality, confirming its importance [4], but the functional implications of the divergence in the TAD domain are largely unknown.

Aberrant increases and decreases in Notch signaling activity are linked to several rare developmental disorders and diverse human cancers, consistent with the important role of Notch as a pleiomorphic developmental regulator [1]. Immature pre-T cells are particularly susceptible to transformation by excessive Notch signaling, as more than 50% of T cell acute lymphoblastic leukemias (T-ALL) derived from these cells have mutations causing ligand-independent NOTCH1 activation [5]. In addition, transduction of ICN1 or “gain of function” human NOTCH1 mutants into murine hematopoetic stem cells induces T-ALL, recapitulating the human disease [6, 7]. The strong association of *NOTCH1* mutations with T-ALL likely reflects key functions of Notch during T cell development, which fails in the absence of *Notch1* and occurs ectopically in the bone marrow when Notch is overactive in hematopoietic progenitor cells [7, 8].

Like *Notch1* and *Notch2*, *Notch3* also is expressed in hematopoietic progenitors and can partially substitute for *Notch1* in T cell lineage specification [9]. In addition, transgenic mice expressing ICN3 develop T-ALL with high penetrance [10], establishing the leukemogenic potential of *Notch3*. Increased NOTCH3 signaling activity in human cells also can be oncogenic; the human cell line TALL1, which has wild-type *NOTCH1* but exhibits sensitivity to gamma secretase inhibitors (GSI; [5, 11]), has a mutation in the NOTCH3 NRR that leads to ligand-independent NOTCH3 activation [11]. This mutation is analogous to previously described activating NOTCH1 mutations in human T-ALL, suggesting that TALL1 is a NOTCH3-dependent, NOTCH1-independent T-ALL cell line.

Here, we use the NOTCH3-mutated T-ALL cell line TALL1 to determine how the genomic response to NOTCH3 compares with the response to NOTCH1 in the NOTCH1-mutated T-ALL cell line CUTLL1. Despite substantial differences in the sequences of NOTCH1 and NOTCH3, particularly within the TAD region, comparative analysis of the genomic landscape of Notch binding sites and of the transcriptional response to activated Notch shows that the oncogenomic effects of NOTCH3 and NOTCH1 in T-ALL cells are highly overlapping. These shared features, including the direct induction of sentinel Notch targets like *NRARP*, as well as subtle differences in the regulatory elements near certain key genes, particularly *MYC*, further refine our understanding of the regulatory networks involving Notch that drive T-ALL cell growth and survival.

## Results

### TALL1 cells are NOTCH3-dependent

**Fig 1:**
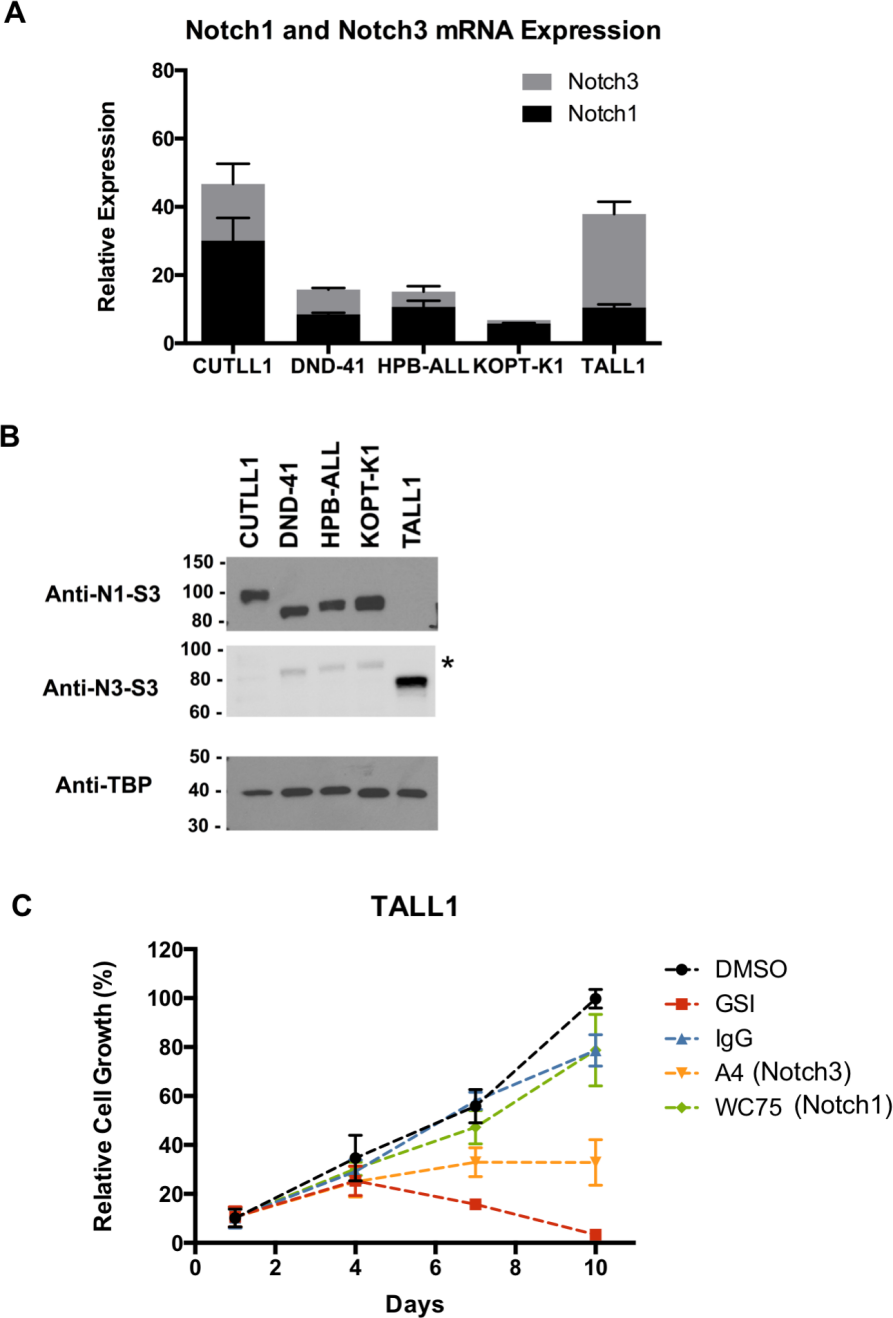
TALL1 cells are NOTCH3-dependent. (A) NOTCH1 and NOTCH3 mRNA transcript levels. Transcripts were quantified using gene specific primer sets and GAPDH as a reference gene. (B) Active nuclear ICN1 and ICN3. Western blots of fractionated cell lysates were stained with the indicated specific antibodies. The anti-N3-S3 antibody, which recognizes gamma-secretase cleaved NOTCH3, has weak cross-reactivity to gamma-secretase cleaved NOTCH1 (asterisk). (C) TALL1 cell growth is strongly inhibited by GSI, partially inhibited by the anti-NOTCH3 NRR antibody A4, and resistant to the anti-NOTCH1 NRR antibody WC75.

TALL1 is one of several T-ALL cell lines that undergo growth arrest in response to treatment with gamma-secretase inhibitors (GSIs; [5]). Unlike other GSI-sensitive cell lines, which harbor activating mutations of NOTCH1, TALL1 cells carry an S1580L mutation in the NOTCH3 NRR that results in ligand-independent NOTCH3 proteolysis [11]. Analysis of mRNA in TALL1, CUTLL1, DND41, HPB-ALL, and KOPTK1 showed that *NOTCH3* and *NOTCH1* mRNAs are expressed in all five cell lines (**Fig 1A**). However, Western blotting with antibodies specific for the gamma-secretase products ICN1 and ICN3 revealed that only TALL1 cells produce ICN3. By contrast, the other four lines produce ICN1, whereas TALL-1 cells do not (**Fig 1B**). These data confirm that NOTCH3 is the source of active Notch signaling in TALL1 cells.

To further link TALL1 cell growth to NOTCH3, we treated these cells with antibodies that selectively block NOTCH1 or NOTCH3. We observed that TALL1 growth is partially inhibited by the anti-NOTCH3 NRR antibody A4 [12], as judged by comparison to the effect of GSI, but was completely unaffected by the anti-NOTCH1 NRR antibody WC75 (**Fig 1C**). The partial growth inhibition seen in the A4-treated cells appears to result from incomplete Notch pathway blockade, as qPCR analysis of the canonical Notch target genes *HES4* and *DTX1* revealed that expression of these genes is strongly suppressed by GSI treatment and more modestly inhibited by A4 treatment (**S1 Fig, related to Fig 1**). Other selective NOTCH3 inhibitory antibodies also suppress TALL1 cell growth [13], further confirming that TALL1 cells are dependent on NOTCH3.

### NOTCH3 and NOTCH1 have similar effects on gene expression in T-ALL cells

**Fig 2:**
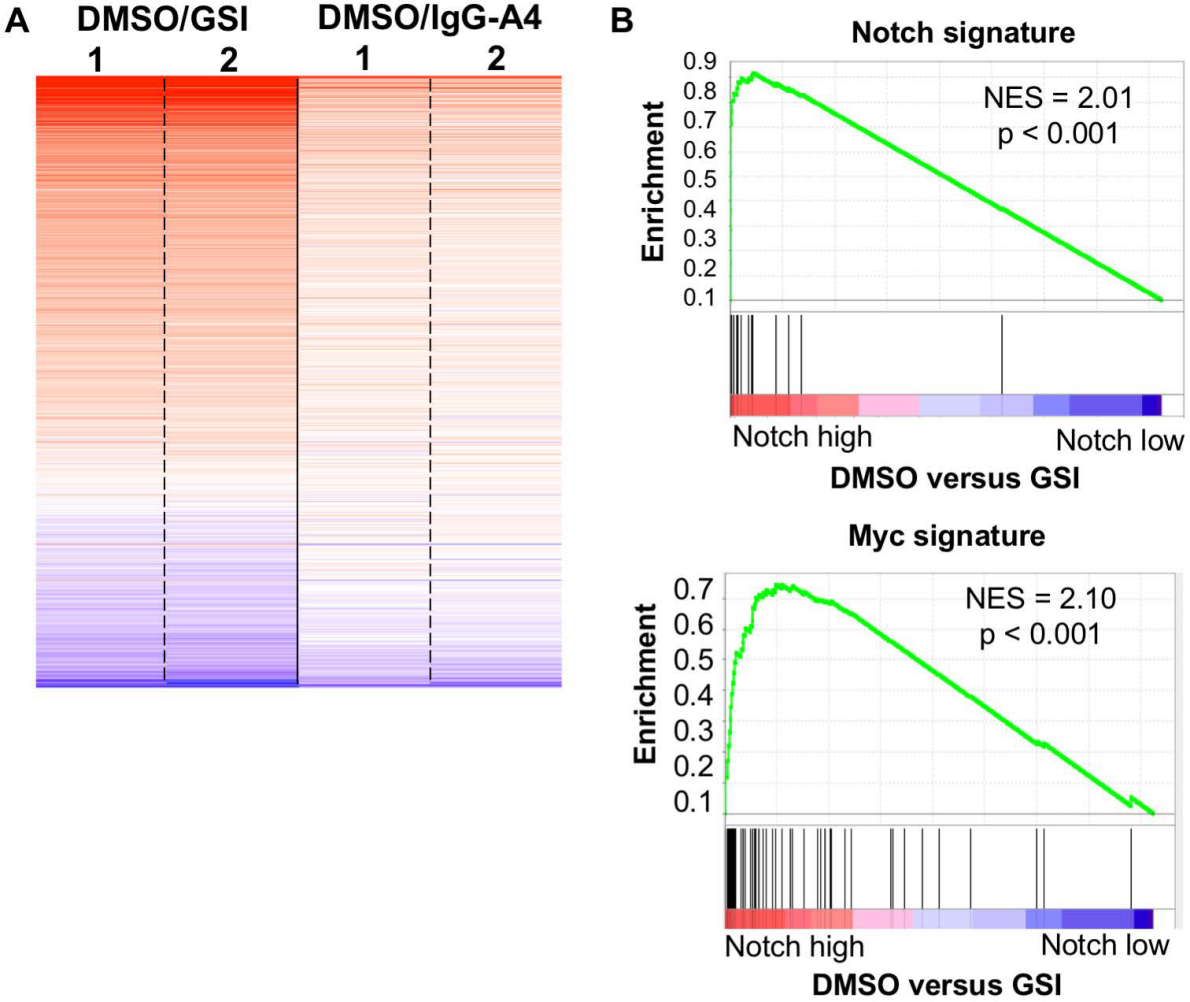
Effects of NOTCH 3 inhibition on TALL1 gene expression. (A) GSI and A4, a NOTCH3-specific blocking antibody, perturb the same gene sets, but to different extents. The fold change in mRNA levels of duplicate samples of TALL1 cells, comparing Notch-on (DMSO) versus Notch-off (either GSI, left or A4 antibody, right) conditions, is shown as a heat map on a log_2_ scale (p<0.05 in either gene set, n = 760). (B) Gene set enrichment analysis (GSEA). Genes showing significantly increased expression in the Notch on state are highly enriched for the Notch1 signature derived from analysis of T-ALL cell lines and for the GSEA Myc signature.

To examine the global effects of NOTCH3 on the transcriptome of TALL1 cells, we performed gene expression profiling of TALL1 cells in Notch “on” and “off” states. Microarray analysis of RNA harvested from cells treated for 24 hours with the GSI compound E, the anti-NOTCH3 antibody A4, or DMSO (vehicle control) revealed that most genes inhibited by GSI were also inhibited by the A4 antibody, but to a lesser extent. Since the A4 antibody is highly specific, this overlap helps to confirm that GSI effects on gene expression in T-ALL cells is largely due to NOTCH3 inhibition (**Fig 2A**). We then used this newly generated TALL1 gene expression data to perform gene-set enrichment analysis (GSEA) with previously defined signatures for NOTCH1 in T-ALL cells and for MYC. This analysis showed that the NOTCH3-on state in TALL1 is highly enriched for Notch and Myc signatures (**Fig 2B**), indicating that NOTCH3 and NOTCH1 have similar effects on the transcriptomes of T-ALL cells.

**Fig 3:**
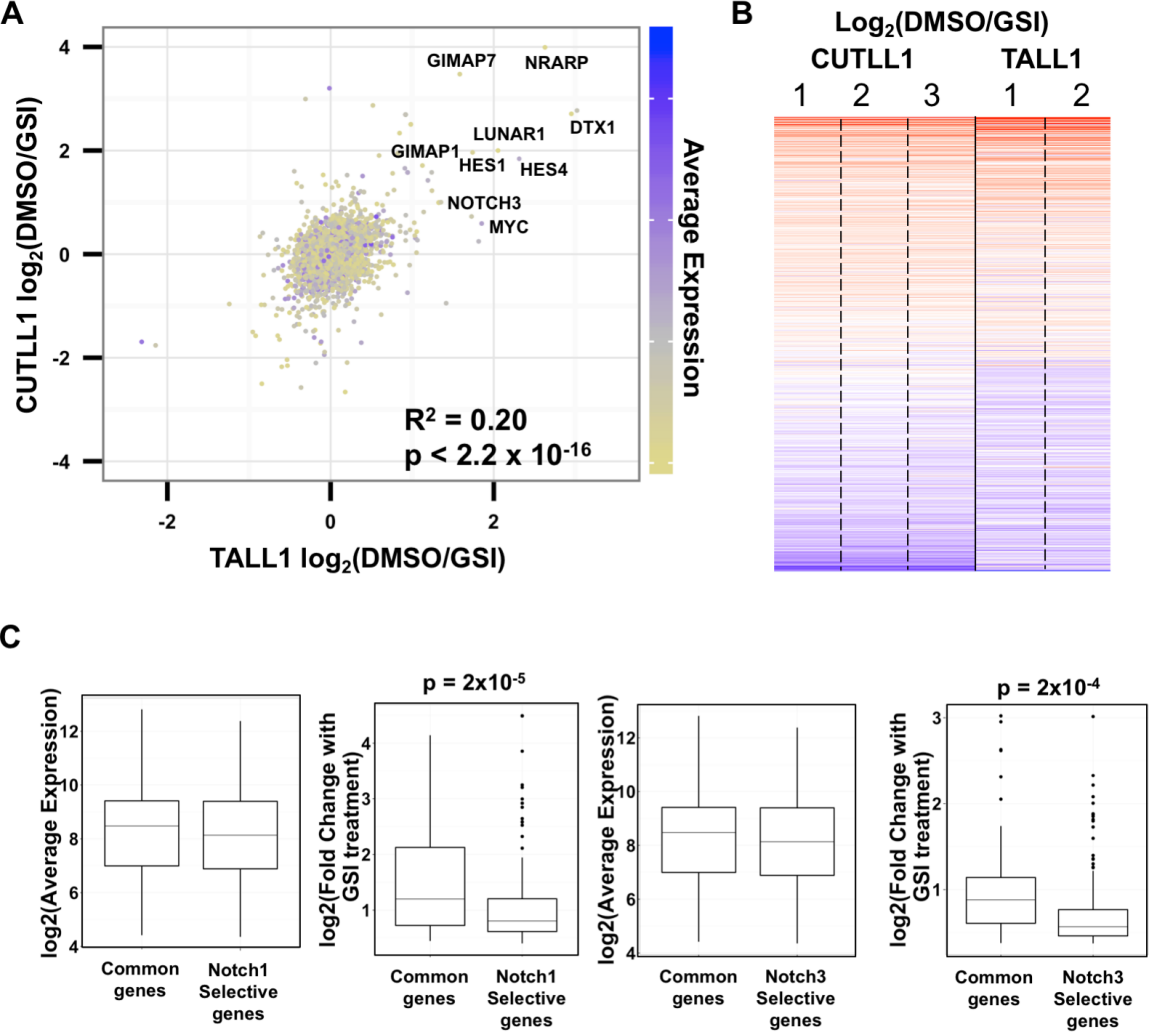
Overlap of NOTCH1- and NOTCH3-regulated genes. (A) Comparison of Notch responsive changes in gene expression between CUTLL1 and TALL1 cell lines. Genes shown are in the top quintile of gene expression, averaged across all samples and represented on a sliding color scale from yellow (low expression) to blue (highest expression). Known NOTCH1 target genes (as indicated) are dynamically regulated in both CUTLL1 and TALL1 cells. (B) Heat map showing genes that exhibit significant changes (p<0.05) in expression between Notch-on and Notch-off states. Genes represented in the heat map are the union of all Notch-dependent genes in both cell lines (n = 1291). The heatmap is ordered vertically by the average fold change in expression across all five rows. (C) Whisker plots comparing average expression and fold-change of genes responding to both NOTCH1 and NOTCH3 (common genes) or selectively to either NOTCH1 or NOTCH3 (selective genes). Though common genes and NOTCH1- and NOTCH3-selective genes have similar overall levels of expression, common genes have a significantly larger fold change in response to GSI treatment. P values were calculated by t-test.

To further evaluate these similarities, we compared the Notch-responsive patterns of gene expression in NOTCH1-dependent CUTLL1 cells and NOTCH3-dependent TALL1 cells. We found that the fold-change of individual genes in response to toggling between Notch-off and -on states showed a strong, statistically significant correlation, which is most easily visualized for the top quintile of all expressed genes (**Fig 3A**). Notable highly correlated examples include the genes *NRARP*, *DTX1*, *HES4*, and *HES1* as well as the non-coding RNA *LUNAR1* [14]. A heat-map of all genes significantly altered by Notch further highlights the similarity of gene regulation by Notch in CUTLL1 and TALL1 cells (**Fig 3B**).

In addition to shared genes, we also identified genes that were significantly altered by Notch activity in only one of the two lines. Genes selectively up-regulated by NOTCH1 were transcribed at the same levels as shared genes that were up-regulated by both NOTCH1 and NOTCH3; however, shared genes had a significantly larger response to Notch than did genes that were induced solely by NOTCH1 or NOTCH3 (**Fig 3C**). Thus, genes that are highly sensitive to NOTCH1 are highly sensitive to NOTCH3 and *vice versa*.

### Comparison of NOTCH3 and NOTCH1 binding to T-ALL cell genomes

**Fig 4:**
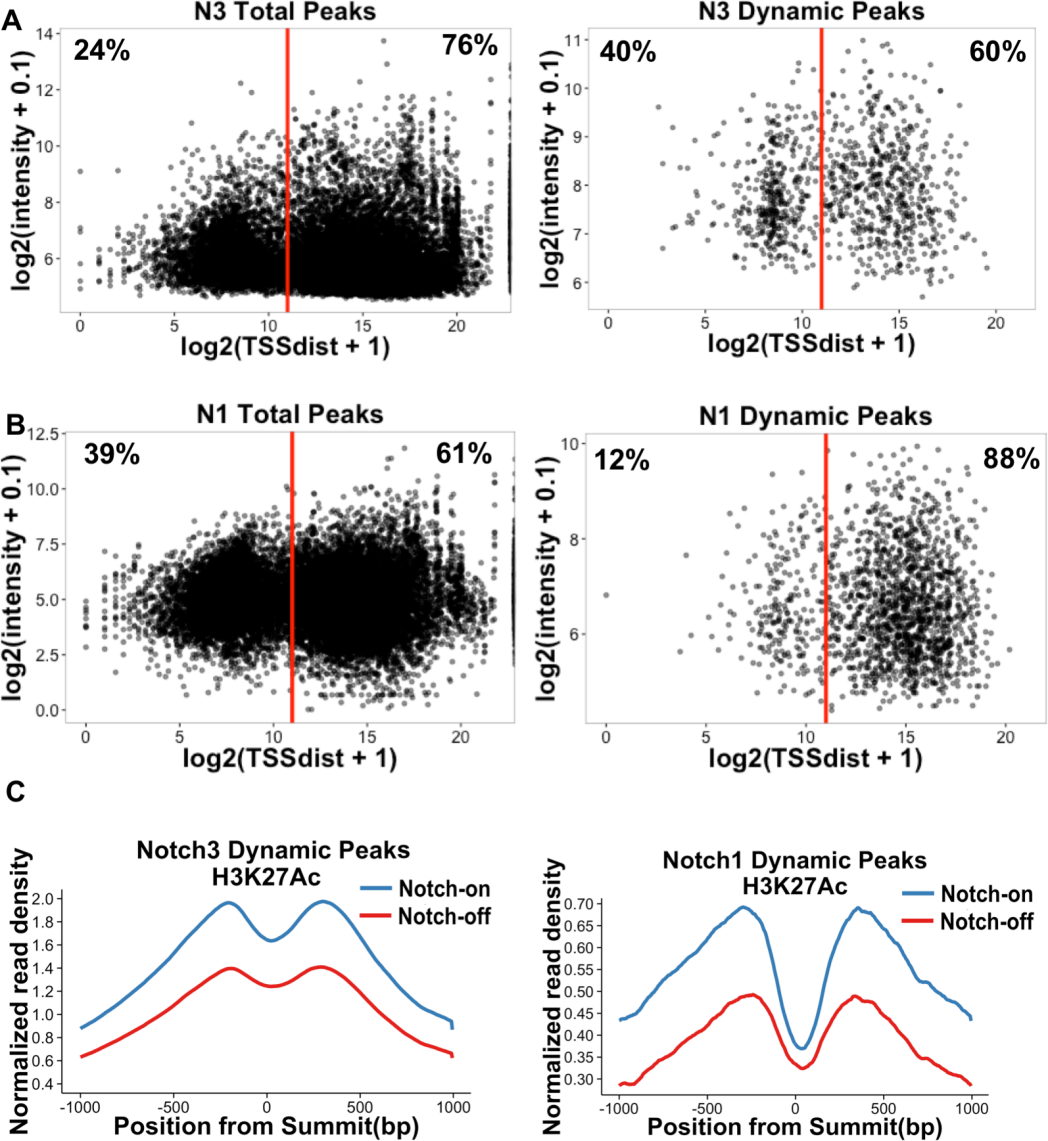
Notch binding to TALL1 and CUTLL1 genomes. (A) Genomic distribution of all NOTCH3 peaks (n = 20577) and dynamic NOTCH3 peaks (n = 992) in TALL1 cells. The graphs show each peak as a function of its distance from the nearest annotated TSS by the total ChIP-seq intensity under the peak. The red line represents 2kb from a TSS, which is the cutoff used for assigning a peak to a promoter or an enhancer. (B) Genomic distribution of all NOTCH1 peaks (n = 17315) and dynamic NOTCH1 peaks (n = 1650) in CUTLL1 cells, plotted in a similar fashion to (A). (C) Across all dynamic NOTCH3 (left) and NOTCH1 (right) peaks, there is a decrease in the normalized read density of H3K27 acetylation in the Notch-off state. The valley in both graphs is centered over the summit (0 bp) of the corresponding NOTCH1 and NOTCH3 peaks.

To determine the genomic sites through which NOTCH3 regulates transcription in TALL1 cells, we performed ChIP-Seq with a NOTCH3-specific antibody and compared the results to previous NOTCH1 data generated in CUTLL1 cells. We found that high confidence NOTCH3 binding sites have a genomic distribution similar to those of NOTCH1 binding sites, with a majority of sites located in enhancers (>2kb from an annotated TSS) (**Fig 4A and 4B**). In addition, the local binding patterns of NOTCH1 and NOTCH3 are nearly identical for certain well-characterized direct Notch target genes, such as the regions around *DTX1*, *NRARP*, and *PTCRA* (**S2 Fig, related to Fig 4**).

Wang *et al*. [15] showed that functional NOTCH1 binding sites that regulate gene expression are characterized by rapid (dynamic) changes in Notch occupancy and H3K27ac when cells are toggled between the Notch-on and -off states. Therefore, to identify functional NOTCH3 binding sites, we performed ChIP-Seq for NOTCH3 and H3K27ac in the Notch-on and -off states, and also re-analyzed prior CUTLL1 cell data sets to generate lists of empirically determined dynamic peaks using a false discovery rate of 0.05 (see methods). Like dynamic NOTCH1 binding sites in CUTLL1 cells, dynamic NOTCH3 sites in TALL1 cells were more common in enhancers than in promoters, although this bias was less pronounced than for dynamic NOTCH1 sites in CUTLL1 cells. As expected, dynamic NOTCH3 peaks in TALL1 cells also were associated with dynamic alterations in H3K27 acetylation (H3K27ac, **Fig 4C**).

**Fig 5:**
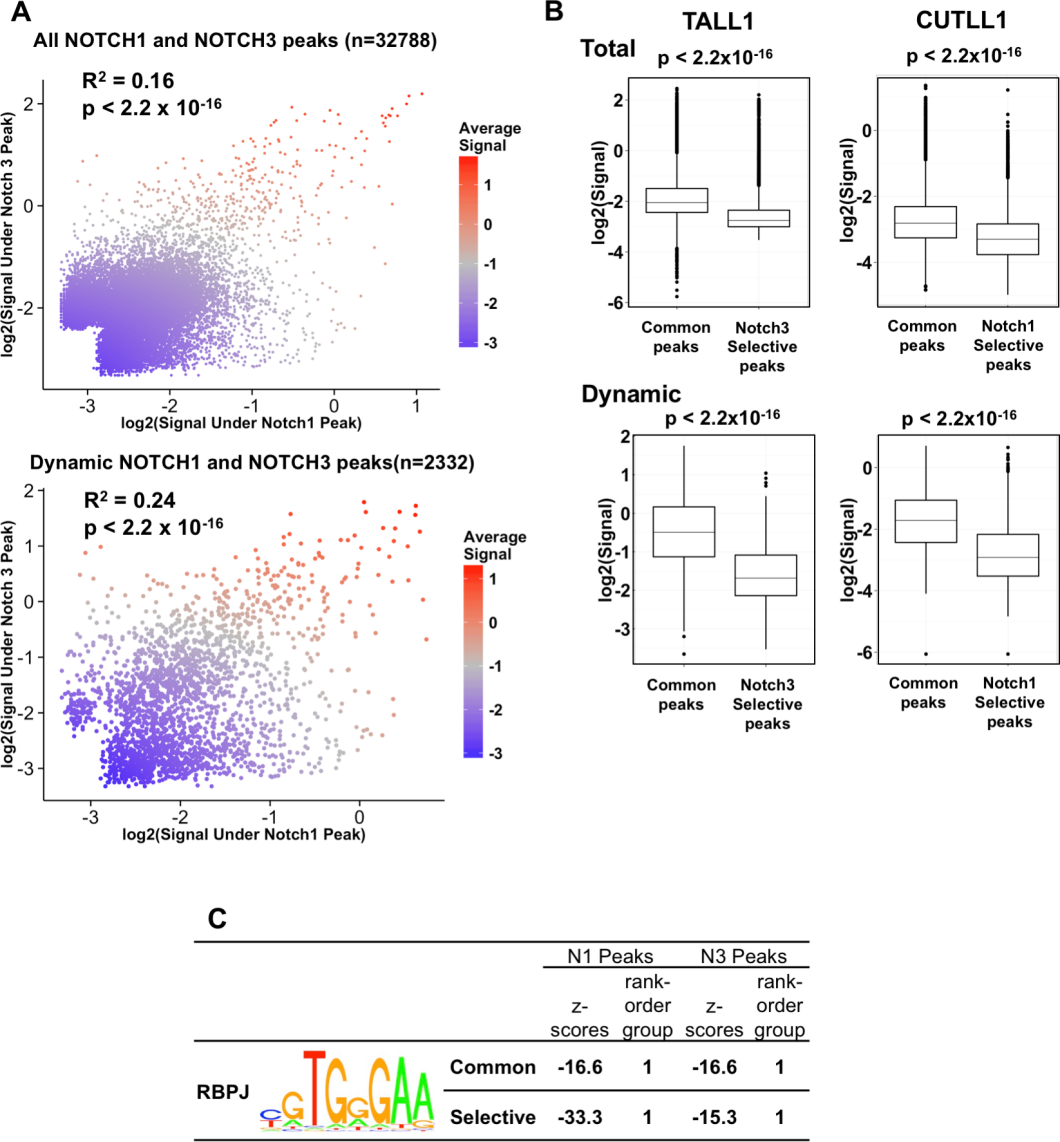
NOTCH1 and NOTCH3 peaks are highly overlapping. (A) Scatter plot showing read count, of total (n = 32788; top) and dynamic (n = 2332; bottom) NOTCH1 and NOTCH3 peaks. The average read count under each peak is indicated in color on a sliding scale from blue (low) to red (high). (B) Whisker plots showing signal strength at NOTCH1 and NOTCH3 shared and selective peaks. Peaks responding to both NOTCH1 and NOTCH3 exhibit much stronger signal strength than NOTCH1 or NOTCH3 selective peaks. (C) Z-scores showing enrichment for the RBPJ motif both at NOTCH1 and NOTCH3 shared peaks and at NOTCH1 or NOTCH3 selective peaks.

To further compare the genomic distribution of functional NOTCH3 and NOTCH1 binding sites, we examined the union of high confidence NOTCH3 and NOTCH1 peaks. We noted a clear correlation between NOTCH3 and NOTCH1 signal intensities when considering either all binding sites or only dynamic binding sites (**Fig 5A**). Of note, sites that bound both NOTCH3 and NOTCH1 showed significantly stronger signal intensities compared to NOTCH3 or NOTCH1 only (“selective”) peaks (**Fig 5B**), and this difference was even greater for dynamic peaks. As with the gene expression analysis, the more NOTCH3 or NOTCH1 binding signal there is at a site, the more likely it is to be bound by the other activated Notch.

### NOTCH3 and NOTCH1 bind at the same *RBPJ* motif locations

To better understand the nature of different types of genomic binding sites, we performed motif analysis on the common dynamic Notch peaks and the selective NOTCH3 or NOTCH1 peaks. The most highly enriched binding motif among the common peaks was that for RBPJ (**Fig 5C**), followed by that for RUNX1. The strong association with *RBPJ* motifs is expected, as it is responsible for binding of activated Notch to genomes. The enrichment for *RUNX* motifs near functional RBPJ/NOTCH1 sites also has been reported in T-ALL cells [16] and likely serves as a mechanism to integrate RUNX and Notch inputs during blood and immune cell differentiation, in line with prior reports of functional interactions between Runx factors and Notch in fish [17], flies [18], and mice [19]. We surmise that common dynamic RBPJ/Notch sites [15] thus include most of the binding sites that are functionally important in maintaining the Notch-dependence of T-ALL cells.

### NOTCH3 and NOTCH1 show divergent binding patterns near a subset of functionally important Notch target genes, including *IL7R*, *IGFR1*, and *MYC*

The overall binding pattern and regulation of gene expression by NOTCH3 in TALL1 cells and NOTCH1 in CUTLL1 cells is remarkably similar (**S2 Fig, related to Fig 5**). However, *IL7R* and *IGFR1*, two Notch target genes reported to be functionally important in NOTCH1-driven T-ALL [20, 21], exhibit strikingly different binding profiles. Specifically, activated NOTCH3 in TALL1 cells does not bind to the 3’ enhancers that have been implicated in NOTCH1-mediated regulation of *IL7R* and *IGFR1* in CUTLL1 cells, suggesting that any difference in *IL7R* or *IGFR1* expression in TALL1 cells results from an indirect effect of agents that perturb Notch activity (**S3 Fig, related to Fig 5**).

**Fig 6:**
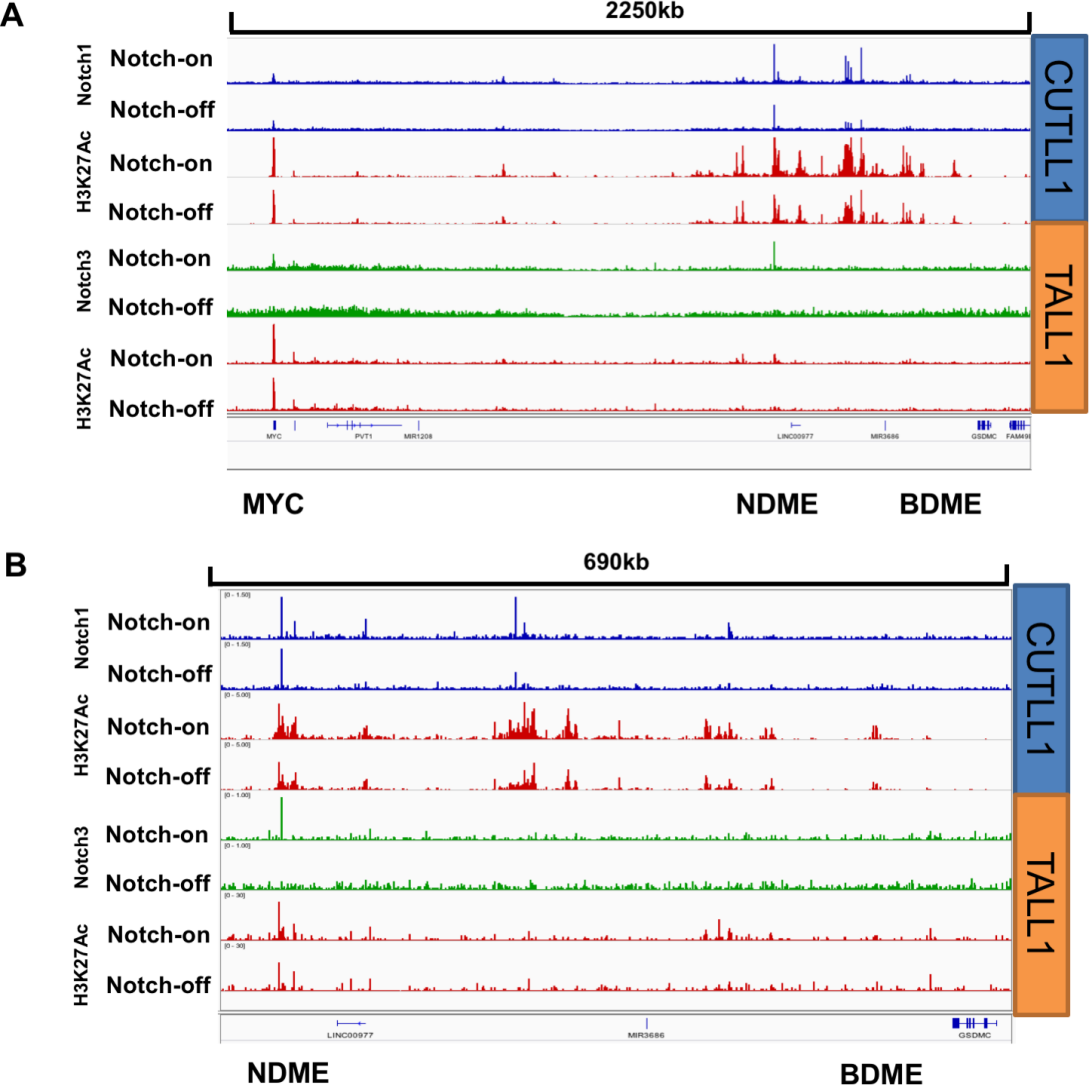
Cell line-specific differences in the *MYC* 3’ enhancer region. (A, B) ChIP-seq signals near *MYC* in the Notch-on and Notch-off states. Panel A shows the entire region from the *MYC* promoter to the BDME region, while panel B zooms in on the region encompassing the NDME and BDME. Signal intensity is normalized for total number of reads and each track pair (Notch-off/Notch-on) is scaled to the same intensity.

**Fig 7:**
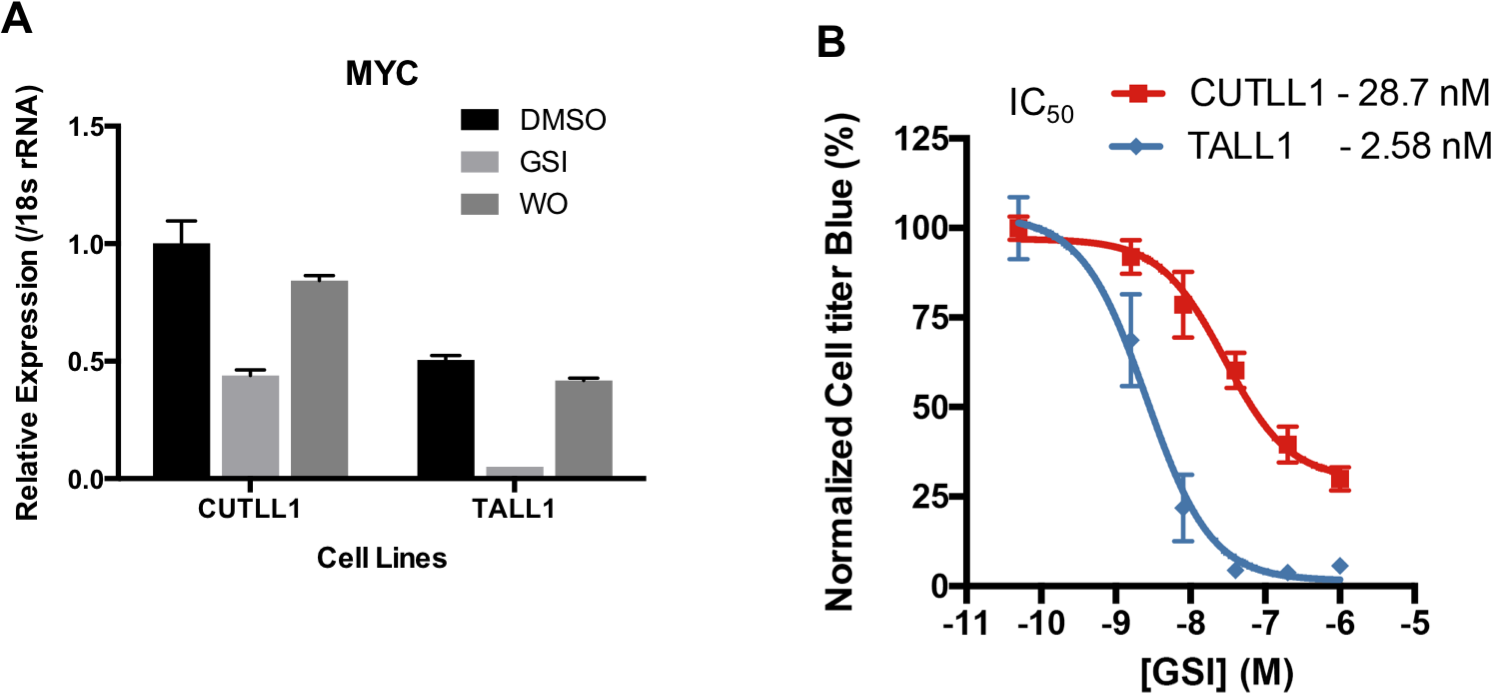
Comparison of the effect of GSI on *MYC* expression and cell growth in TALL1 cells and CUTLL1 cells. (A) qPCR analysis of *MYC* mRNA, normalized relative to 18s rRNA, in CUTLL1 cells and TALL1 cells. Cells were treated with DMSO or GSI for 3 days and incubated for 24 hours after GSI washout. (B) TALL1 is more sensitive to Notch inhibition than CUTLL1 cells. Cell viability, assessed using cell titer blue, was measured as a function of the dose of compound E.

A second intriguing difference between the NOTCH1 and NOTCH3 ChIP-Seq results concerns the *MYC* locus. In prior studies of NOTCH1-dependent T-ALL cells, *MYC* expression has been linked to a large 3’ enhancer region denoted by a high content of acetylated H3K27 that contains a Notch-dependent MYC enhancer (NDME) module and a more 3’ BRD4-dependent MYC enhancer (BDME) module [22, 23]. Notably, in contrast to other T-ALL cell lines such as CUTLL1, which have high levels of H3K27ac in both the NDME and the BDME, in TALL1 cells the H3K27ac signal in this region is attenuated by comparison, and most of the H3K27ac marks that are present are confined to the region near a dynamic NOTCH3 binding site in the NDME (**Fig 6**). Additional weak Notch peaks that are seen 3’ of the NDME in CUTLL1 cells are absent in TALL1 cells, and regions corresponding to the BDME show little H3K27 acetylation. The strong Notch3 peak in the NDME and the restriction of H3K27ac marks to the *MYC* promoter and to regions near the site of NOTCH3 binding in the NDME suggested that *MYC* expression in TALL1 cells might be more dependent on Notch than it is in CUTLL1 cells, where the BDME also is active and loops to the *MYC* gene body [23]. Consistent with this idea, the suppression of NOTCH3 activity with GSI greatly decreases *MYC* expression in TALL1 cells, while GSI treatment of CUTLL1 cells reduces *MYC* expression by only ~50% (**Fig 7A**). Of further note, expression of *MYC* in CUTLL1 cells is higher than in TALL1 cells, a second difference that also may be related to different enhancer states in these two lines. Given the importance of MYC in the growth of Notch-“addicted” T-ALL cells, it is also not surprising that TALL1 cells are approximately 10-fold more sensitive to GSI than are CUTLL1 cells (**Fig 7B**).

### NOTCH1 and NOTCH3 are functionally interchangeable in T-ALL cells

**Fig 8:**
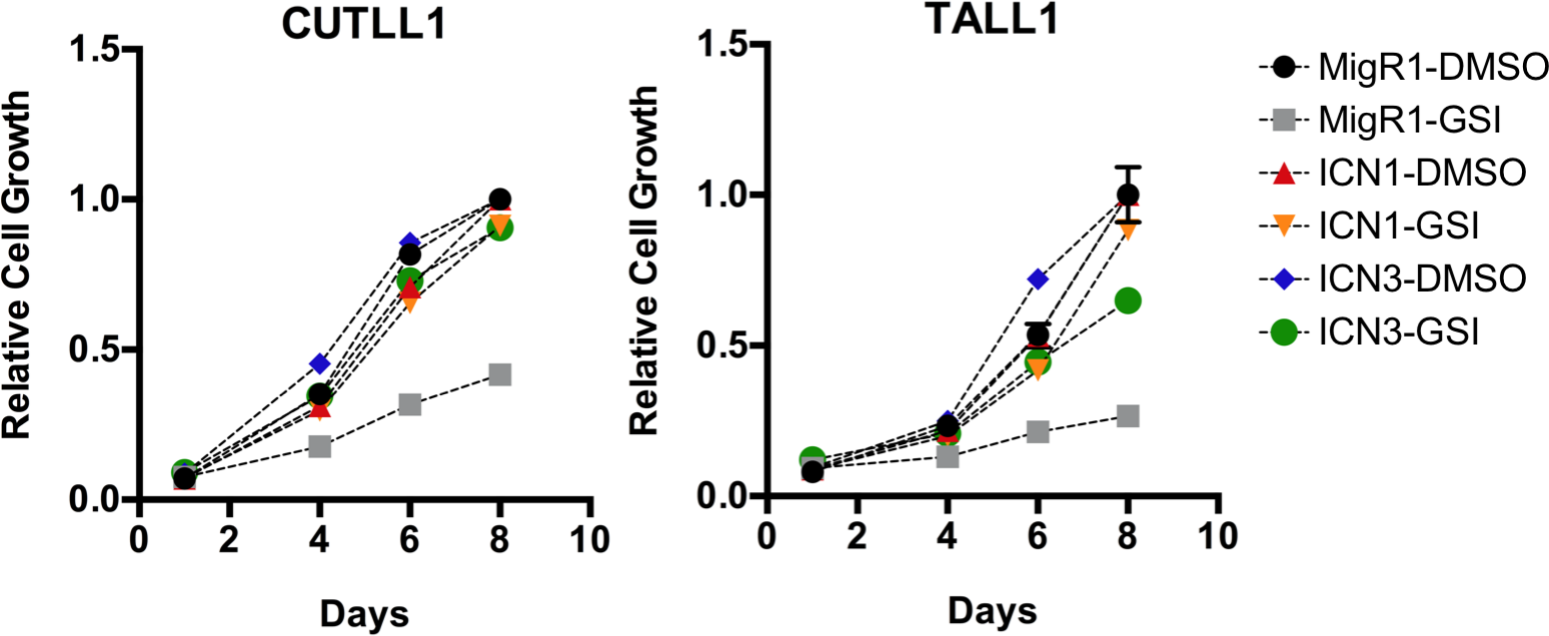
Interchangeability of ICN1 and ICN3 in TALL1 cells and CUTLL1 cells. Cell growth plotted as a function of time under different treatment conditions, showing rescue of GSI treated TALL1 and CUTLL1 cells by either ICN1 or ICN3.

The differences in *MYC* expression and regulation cited above could stem from cell line specific differences in enhancers or could conceivably stem from a functional difference between NOTCH1 and NOTCH3. To confirm that NOTCH1 can support the growth of TALL1 cells and that NOTCH3 can support the growth of CUTLL1 cells, we introduced ICN1 and ICN3 into CUTLL1 and TALL1 cell lines using retroviral transduction, and monitored cell growth in the presence of the GSI compound E, which inhibits signaling from endogenous Notch polypeptides but not ICN. We found that ICN1 or ICN3 were competent to rescue both CUTLL1 and TALL1 cells from the effects of GSI (**Fig 8** and **S4 Fig, related to Fig 8**). This finding suggests that despite substantial sequence divergence, ICN1 and ICN3 are effectively interchangeable in their ability to drive the expression of downstream target genes that support T-ALL cell growth.

## Discussion

NOTCH1 is a well-known oncogenic driver of T-ALL, with NOTCH1-activating somatic mutations present in approximately 60% of cases. The availability of a leukemic cell-line driven by an activating mutation in NOTCH3 affords an opportunity to assess whether there is a common oncogenomic program shared by different activated Notch receptors.

Our data argue that NOTCH3 and NOTCH1 drive T-ALL oncogenesis through similar pathways, as assessed both by global changes in gene expression and by genome-wide analysis of DNA sites bound by ICN. Changes in gene expression between the Notch-on and Notch-off states are correlated between the two cell lines, especially for genes that are highly responsive to the effects of either receptor. Whereas shared genomic Notch1 and NOTCH3 binding sites clearly identify functionally important regulatory elements, the functional significance of elements that selectively bind NOTCH1 or NOTCH3 remains less clear.

Despite a number of potentially confounding variables in our experimental design, including differences in the genetic backgrounds of the cell lines under study, there remains a remarkable spectrum of similarities between the targets of NOTCH3 and NOTCH1, indicating that there is a robust shared common pathway between Notch-“addicted” leukemias. Many genes that are directly regulated by both NOTCH1 and NOTCH3 are well-established Notch targets, including *NRARP*, *DTX*1, *HES1*, *HES4*, *PTCRA*, and *MYC*, along with the non-coding RNA *LUNAR1*.

There are, nevertheless, context-specific differences between the two cell lines. CUTLL1 cells have a p53 mutation, which may influence the responsiveness of these cells to activated Notch1 [24]. Conversely, studies in transgenic mice have implicated Notch3 specifically in the functional inactivation of Ikaros [25]. The most striking distinction between the two cell lines, however, resides in the chromatin landscape around *MYC*.

*MYC* is the most prominent Notch target gene in NOTCH1-dependent T-ALL, and its contribution to T-ALL cell proliferation and tumor maintenance is well established [26, 27]. NOTCH1-dependent T-ALL cell lines (e.g., CUTLL1, DND-41, and KOPT-K1) have RBPJ/Notch binding sites 1.3 Mb 3’ of the *MYC* gene body that are associated with a broad multidomain enhancer defined by H3K27 acetylation marks and binding of the BET bromdomain protein BRD4 [23, 28]. This site, which loops back to interact with the *MYC* promoter in CUTLL1 cells, has been termed the Notch-dependent *MYC* enhancer, or NDME. In the face of long-term inhibition of Notch signaling by GSI, resistant T-ALL cells emerge in which a nearby BRD4-dependent *MYC* enhancer element (BDME) interacts with the *MYC* promoter and drives *MYC* expression [22, 23].

The chromatin landscape around the *MYC* locus in TALL1 cells, however, differs from that seen in these other NOTCH1-dependent T-ALL cell lines [22, 23]. Whereas the region 3’ of *MYC* shows one major and several minor NOTCH1 binding sites and an extended zone of H3K27ac marks encompassing both the NDME and BDME in both humans and mice, in TALL1 cells, NOTCH3 binding is observed only in the NDME and the deposition of H3K27Ac in the region encompassing the NDME and BDME is attenuated, with BDME H3K27ac. The different impact of Notch3 binding on this 3’ *MYC* enhancer NDME in TALL1 cells may explain the unusual sensitivity of TALL1 cells to Notch blockade with GSIs, both in terms *MYC* down-regulation and inhibitory effects on growth. Because ICN1 and ICN3 can substitute for one another in Notch-dependent T-ALL cells and because there is no evidence to date that Notch complexes can bind and open repressed chromatin, we speculate that the observed differences result either from cell-line specific differences in factors operating upstream of Notch that regulate chromatin states, or from variation in the capacity of the divergent “TAD” regions of Notch1 and Notch3 to recruit histone acetyltransferases such as p300 in concert with Mastermind-like co-activators. It will be of interest to determine which of these *MYC* enhancer landscapes occur most frequently in primary human T-ALLs, as they may prove to be predictors of response to Notch-directed therapies such as GSIs.

## Materials and methods

### Cell culture

CUTLL1, DND-41, HPB-ALL, KOPT-K1 and TALL1 cells were cultured in RPMI1640 medium supplemented with 10% FBS and 1x penicillin and streptomycin. 293T cells were maintained in DMEM medium supplemented with 10% FBS.

### Retroviral transduction

Retroviral MigR1-GFP, MigR1-ICN1-GFP and MigR1-ICN3-GFP are described [8, 29]. Retrovirus packaging and transductions were performed as described [30]. In brief, MigR1 constructs were co-transfected with packaging plasmids (VSV-G and gag-pol) into 293T cells using Lipofectamine 2000 (Invitrogen). Packaged viruses were harvested after 48 hours and cleared by filtering through a 0.45 μm membrane. Retroviral transduction (1 x 10^6^ cells) was performed in the presence of polybrene (4 μg/ml) and GFP expression was monitored by flow cytometry. Two weeks after transduction, viable GFP-positive cells were sorted by FACS (SONY SH800z Cell Sorter).

### Cell growth assay

Cells (5 x 10^3^) were plated in 96 well (Black well, clear bottom) plates and incubated with DMSO or GSI (1 μM). Viable cells were estimated using Cell Titer Blue (Promega). To measure IC50 values, cells were incubated with serial five-fold dilutions of GSI or DMSO for 7 days and viable cell populations were measured as above.

### Quantitative RT-PCR

RNA was extracted using a Qiagen RNeasy kit and cDNA was synthesized with a QuantiTect reverse transcription kit (Qiagen). Quantitative PCR was performed on a Bio-Rad CFX instrument using the SYBR PCR master mix (Bio-Rad). Primers are provided in S1 Table.

### Western blotting

Fractionated cell lysates were prepared using a NE-PER Nuclear and Cytoplasmic Extraction kit (Thermo Scientific) and subjected to Western blotting using anti-NOTCH1 (anti-TAD; [16]), anti-V1744 antibody recognizing S3-cleaved NOTCH1 (Cell Signaling #4147); anti-Notch3 (Cell Signaling #2889); a polyclonal anti-NOTCH3 antibody raised against the neoepitope generated by S3 cleavage of NOTCH3; anti-TBP (Cell Signaling #8515); or anti-GAPDH (Cell Signaling #2118). The polyclonal anti-NOTCH3 antibody recognizing S3-cleaved NOTCH3 was raised against the KLH conjugated peptide epitope (VMVARRKREHSTLWFPG) (Covance) and affinity purified using a peptide epitope-coupled resin.

### Notch perturbation in cultured cells

Previous analyses in CUTLL1 cells used 3 days of treatment with the GSI compound E to achieve a “Notch off” state. Because of the nature of the *NOTCH1* mutation in CUTLL1 cells (REF), GSI blockade results in accumulation of S2-cleaved NOTCH1 at the cell membrane, which is rapidly chased to S3-cleaved ICN1 following GSI washout, allowing changes caused by acute Notch activation to be monitored. By contrast, the exquisite sensitivity of TALL1 cells to GSI (Fig 1C) and significant decrease in total NOTCH3 at the cell surface post GSI treatment precludes reactivation of NOTCH3 by GSI washout in TALL1 cells. We thus assessed the “NOTCH3-off” state after 1 day of GSI treatment, while the “NOTCH3 on” state corresponded to cells treated with vehicle (DMSO) for 1 day. As in prior experiments with CUTLL1 cells, the GSI compound E (1 μM) was used in experiments with TALL1 cells.

### RNA preparation and gene expression array

Total RNA extraction was performed using Trizol reagent (Life Technologies) and RNAeasy kit (Qiagen). Duplicate samples were subjected to expression profiling on Affymetrix Human Genome U133 Plus 2.0 Array. Gene expression data are available through the Gene Expression Omnibus (GEO accession number GSE104262).

### Gene expression analysis

Gene expression analysis was performed on the new data generated for TALL1 cells and prior data for CUTLL1 cells (GEO Series accession GSE29544) beginning with the raw CEL files for both. The CEL files were preprocessed in R [31] to perform RMA normalization. Differential expression between conditions within each cell line was performed using Linear Models for Microarray Data (Limma), with a p value of 0.05 for significance. Figures were produced using ggplot2.

### Gene set enrichment analysis

The Broad Institute GSEA program was used [32, 33]. Gene ordering used the gene ranking metric of log2 ratio of classes and remaining default settings. Gene sets were the 50 Broad Hallmark sets and one previously curated Notch T-ALL gene set [34].

### ChIP-seq

ChIP was performed using EZ-Magna ChIP (Millipore) according to the manufacturers protocol. In brief, cells were fixed, sheared by sonication and immunoprecipitated with rabbit IgG control (supplied in the kit), anti-NOTCH3 antibody (Cell Signaling, #2889), or anti-H3K27Ac antibody (Abcam, ab4729). Libraries were constructed using an NEB NEXT kit, and sequencing was done on an Illumina Hiseq2000 instrument. ChIP-seq data are available through the Gene Expression Omnibus (accession number GSE104262)

### Analysis of ChIP-seq data

ChIP-seq data was aligned to version 38 of the human genome (hg38) using bowtie2 with the default settings [35]. Datasets without a minimum of 50% unique reads were not used for subsequent analyses. Uniquely mapped, non-duplicate reads were processed with macs2 [36], without input controls, using an FDR of 0.01 and default settings. Datasets with reads fractions within peaks of >1% were used for subsequent analyses. Dynamic NOTCH1 and NOTCH3 binding sites were determined as reported previously [15]. Further analysis of ChIP-seq intensities across genomic intervals was performed using Bedtools [37] and UCSC genome browser utilities [38]. ChIP-seq peak distribution across the genome was determined using CEAS v1.0.0 with default options [39]. Motif analysis was performed using SeqPos v1.0.0 with default options [40] ChIP-seq track visualizations were produced with IGV [41]. Other visualizations were produced using ggplot2.

## Supporting information

S1 Fig Effects of the gamma secretase inhibitor Compound E (GSI), inhibitory anti-NOTCH1 antibody (WC75), and inhibitory anti-NOTCH3 antibody (A4) on expression of Notch target genes in T-ALL cell lines. (PDF)

S2 Fig IGV tracks near DTX1, NRARP, and PTCRA in TALL1 and CUTLL1 cells. (PDF)

S3 Fig Notch binding, IL7R and IGF1R enhancer landscapes, and IL7R and IGF1R expression in CUTLL1 and TALL1 cells. (PDF)

S4 Fig Flow cytometric analysis of GFP expression in transduced CUTLL1 and TALL1 cells. (PDF)

S1 Table qPCR primers. (PDF)
