# Supplementary material for: The common oncogenomic program of NOTCH1 and NOTCH3 signaling in T-cell acute lymphoblastic leukemia
Source: PLoS One. 2017 Oct 12;12(10):e0185762. doi: 10.1371/journal.pone.0185762 (PMC5638296; doi:10.1371/journal.pone.0185762)
Supplement: S1 Fig — (PDF) [file pone.0185762.s001.pdf]

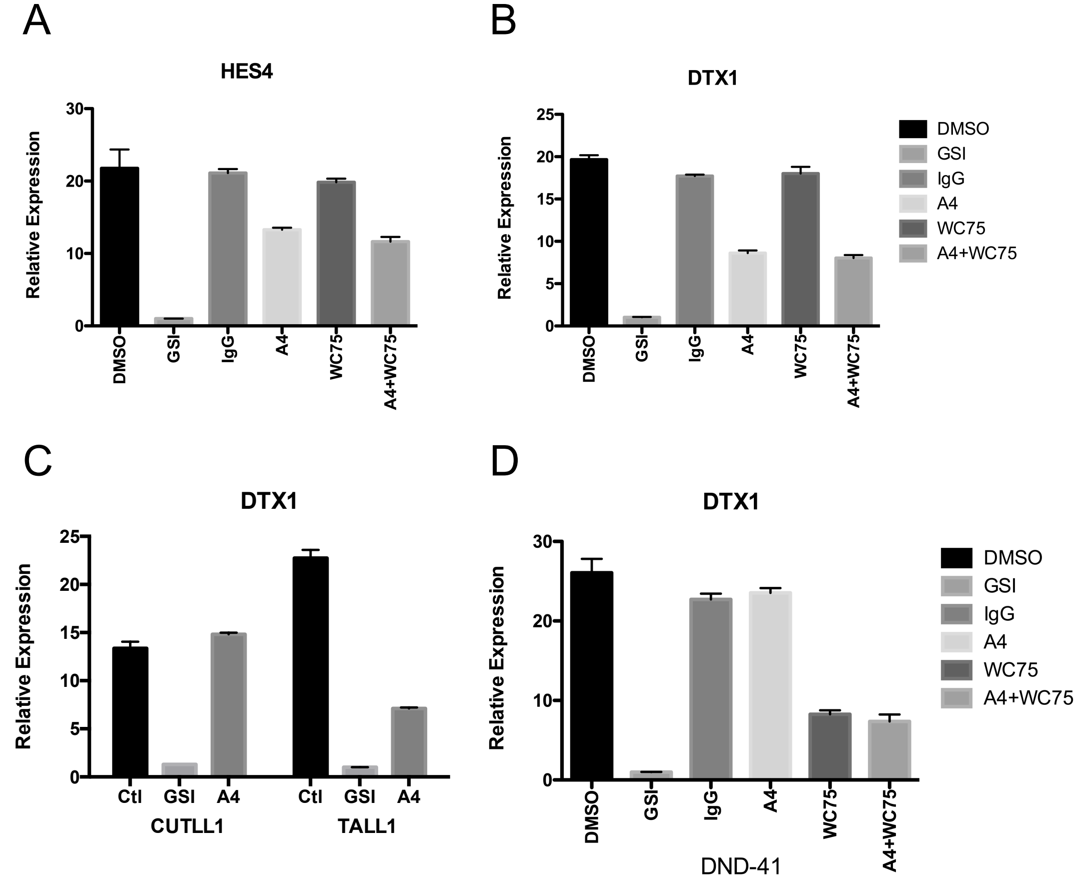

Supplemental Figure 1. Effects of the gamma secretase inhibitor Compound E (GSI), inhibitory anti-NOTCH1 antibody (WC75), and inhibitory anti-NOTCH3 antibody (A4) on expression of Notch target genes in T-ALL cell lines. A. Effect of various inhibitors on *HES4* expression in NOTCH3-mutated TALL1 cells. B. Effect of various inhibitors on *DTX1* expression in TALL1 cells. C. Effects of GSI and A4 treatment on *DTX1* expression in NOTCH1-mutated CUTLL1 cells and TALL1 cells. D. Effects of GSI, WC75, and A4 on *DTX1* expression in NOTCH1-mutated DND-41 cells.
