# Supplementary material for: The common oncogenomic program of NOTCH1 and NOTCH3 signaling in T-cell acute lymphoblastic leukemia
Source: PLoS One. 2017 Oct 12;12(10):e0185762. doi: 10.1371/journal.pone.0185762 (PMC5638296; doi:10.1371/journal.pone.0185762)
Supplement: S2 Fig — (PDF) [file pone.0185762.s002.pdf]

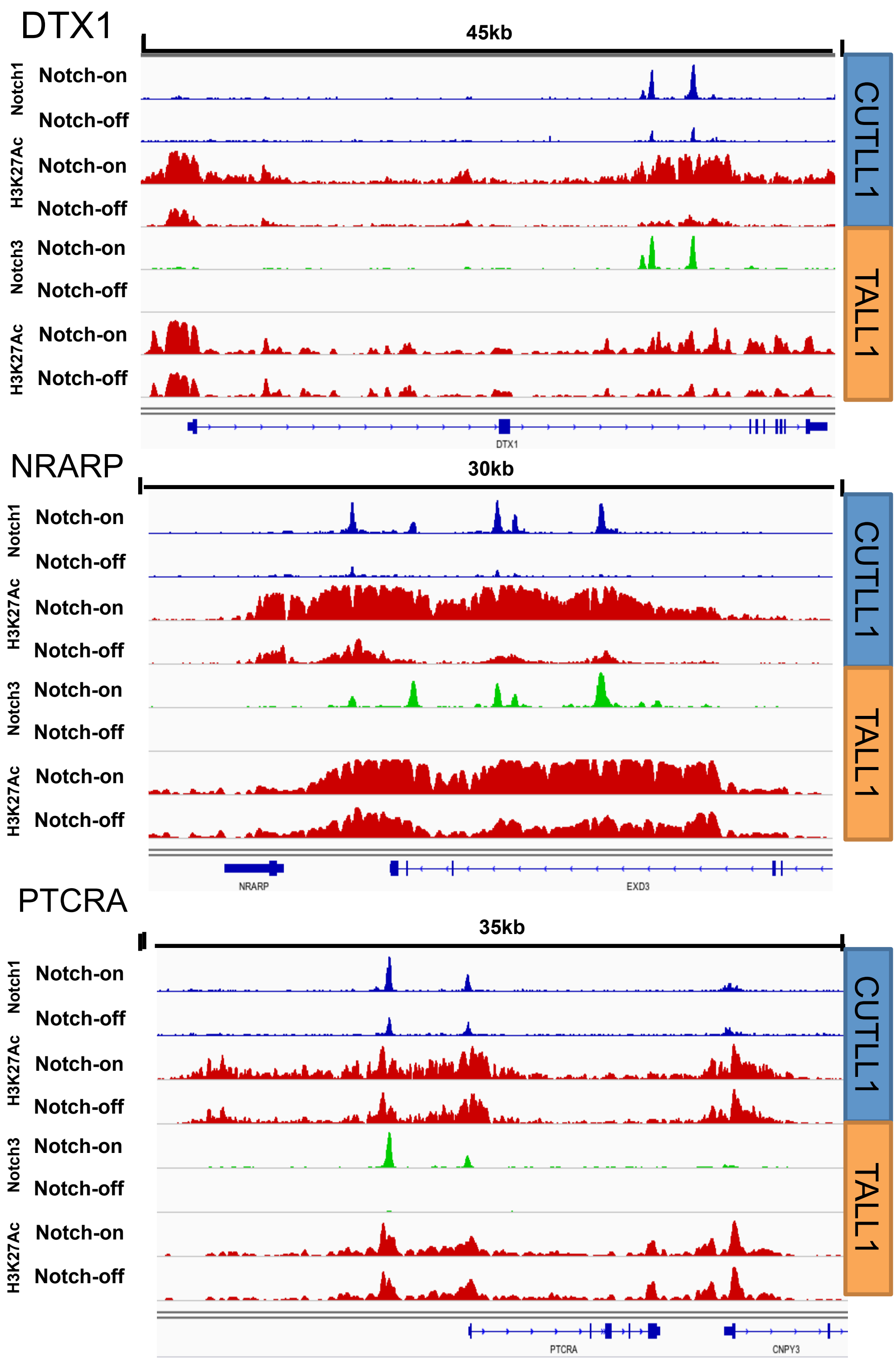

Supplemental Figure 2. IGV tracks showing similar chromatin and Notch-binding chromatin landscapes near *DTX1*, *NRARP*, and *PTCRA* in TALL1 and CUTLL1 cells.
