# Supplementary material for: The common oncogenomic program of NOTCH1 and NOTCH3 signaling in T-cell acute lymphoblastic leukemia
Source: PLoS One. 2017 Oct 12;12(10):e0185762. doi: 10.1371/journal.pone.0185762 (PMC5638296; doi:10.1371/journal.pone.0185762)
Supplement: S3 Fig — (PDF) [file pone.0185762.s003.pdf]

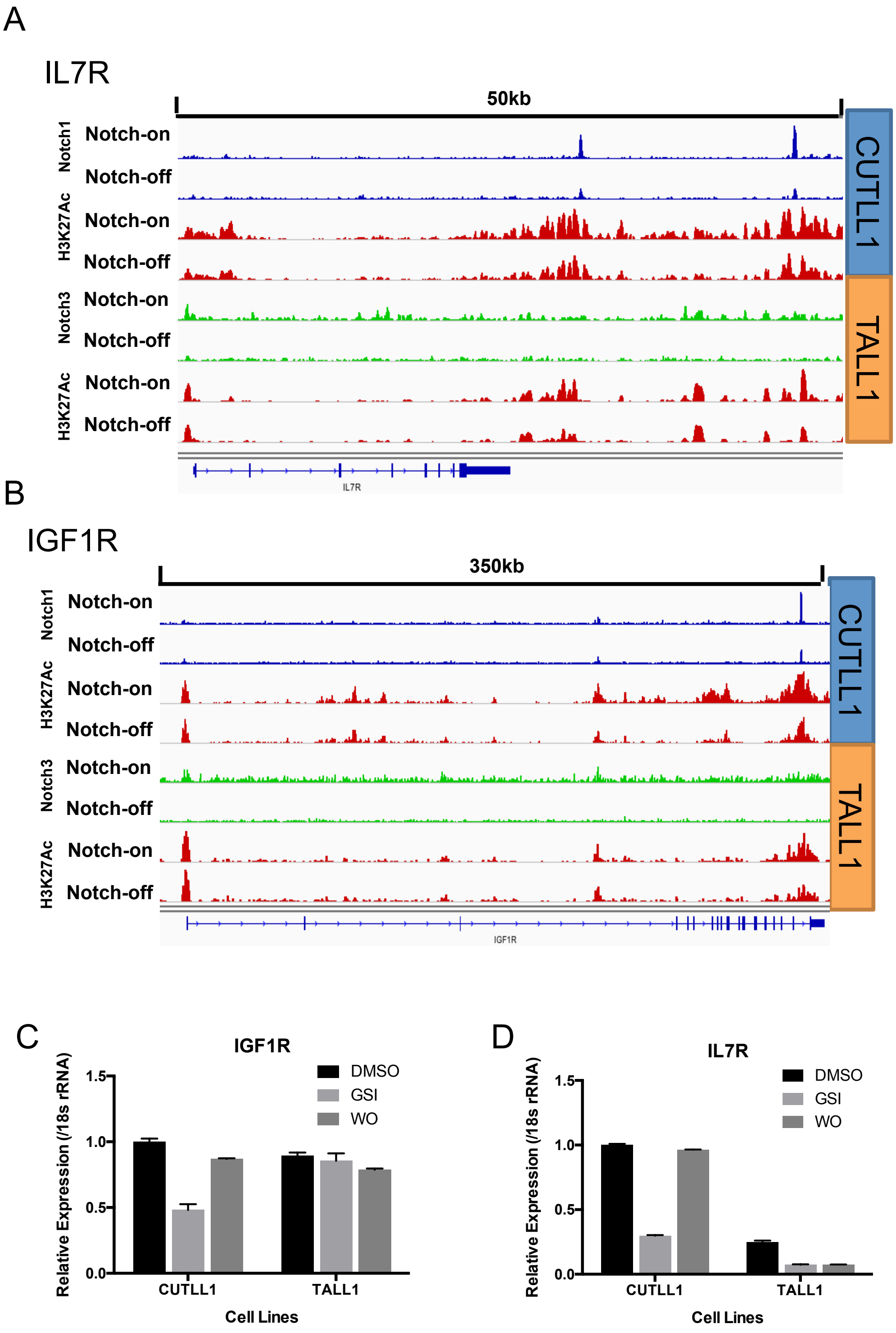

Supplemental Figure 3. Notch binding, *IL7R* and *IGF1R* enhancer landscapes, and *IL7R* and *IGF1R* expression in CUTLL1 and TALL1 cells. A, B. IGV tracks showing NOTCH1 and NOTCH3 binding and H3K27ac chromatin landscapes near *IL7R* and *IGF1R*. C, D. qRT-PCR analysis of *IGF1R* and *IL7R* expression in CUTLL1 and TALL1 cells.
