# Supplementary material for: The common oncogenomic program of NOTCH1 and NOTCH3 signaling in T-cell acute lymphoblastic leukemia
Source: PLoS One. 2017 Oct 12;12(10):e0185762. doi: 10.1371/journal.pone.0185762 (PMC5638296; doi:10.1371/journal.pone.0185762)
Supplement: S4 Fig — (PDF) [file pone.0185762.s004.pdf]

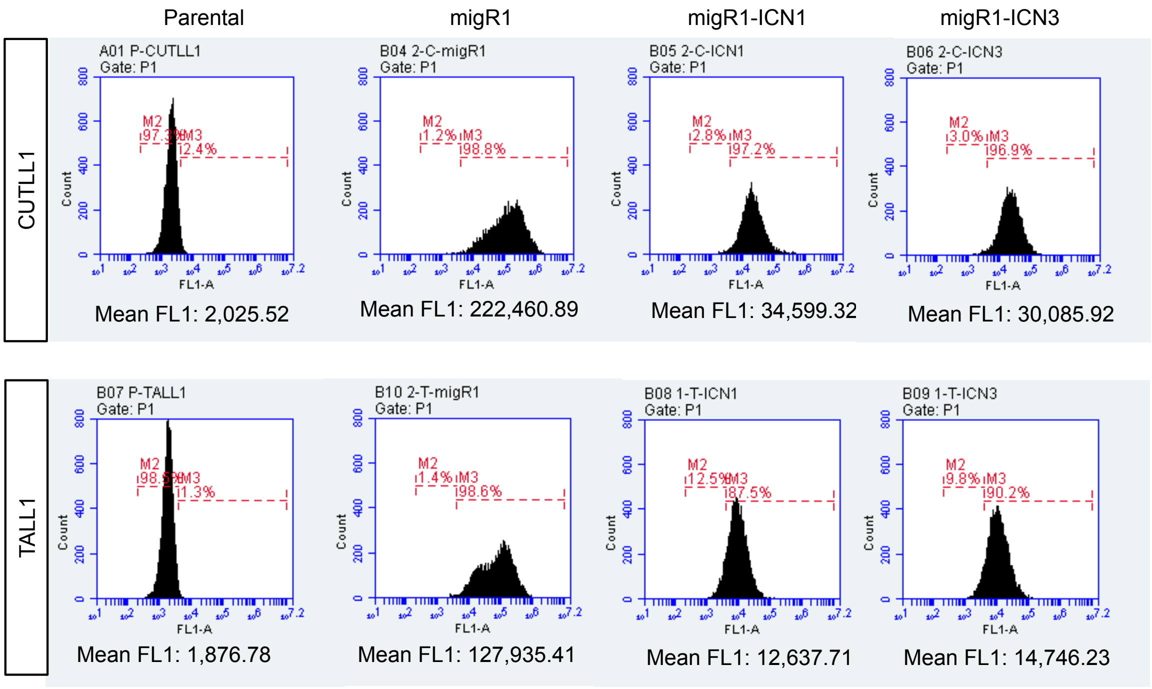

Supplemental Figure 4. Flow cytometric analysis of GFP expression in CUTLL1 and TALL1 cells transduced with the indicated retroviruses.
