# Supplementary material for: The common oncogenomic program of NOTCH1 and NOTCH3 signaling in T-cell acute lymphoblastic leukemia
Source: PLoS One. 2017 Oct 12;12(10):e0185762. doi: 10.1371/journal.pone.0185762 (PMC5638296; doi:10.1371/journal.pone.0185762)
Supplement: S1 Table — (PDF) [file pone.0185762.s005.pdf]

Supplemental Table 1: qPCR primers

		Sequence
Notch1a	F	5' – TGGACCAGATTGGGGAGTTC – 3'
	R	5' – GCACACTCGTCTGTGTTGAC – 3'
Notch1b	F	5' – CAACATCCAGGACAACATGG – 3'
	R	5' – GGACTTGCCCAGGTCATCTA – 3'
Notch3a	F	5' – TGGCGACCTCACTTACGACT – 3'
	R	5' – CACTGGCAGTTATAGGTGTTGAC – 3'
Notch3b	F	5' – GTAGAGGGCATGGTGGAAGA – 3'
	R	5' – AAGTGGTCCAACAGCAGCTT – 3'
MYC	F	5' – GGCTCCTGGCAAAAGGTCA – 3'
	R	5' – CTGCGTAGTTGTGCTGATGT – 3'
GAPDH	F	5' – GAAGGTGAAGGTCGGAGTCAAC – 3'
	R	5' – TGGAAGATGGTGATGGGATTTC – 3'
18s rRNA	F	5' – TAGAGTGTTCAAAGCAGGCCC – 3'
	R	5' – CCAACAAAATAGAACCG CGGT – 3'
Negative Control	F	5' – AATGCTGGGCTTCCAAGGA – 3'
	R	5' – GACCTTGGTGACTGTTGAGGAAAC – 3'
